# The Chikungunya Virus Capsid Protein Contains Linear B Cell Epitopes in the *N*- and *C*-Terminal Regions that are Dependent on an Intact *C*-Terminus for Antibody Recognition

**DOI:** 10.3390/v7062754

**Published:** 2015-06-08

**Authors:** Lucas Y. H. Goh, Jody Hobson-Peters, Natalie A. Prow, Kelly Baker, Thisun B. H. Piyasena, Carmel T. Taylor, Ashok Rana, Marcus L. Hastie, Jeff J. Gorman, Roy A. Hall

**Affiliations:** 1 Australian Infectious Diseases Research Centre, School of Chemistry and Molecular Biosciences, The University of Queensland, St Lucia, Queensland 4072, Australia; E-Mails: l.goh1@uq.edu.au (L.Y.H.G.); j.peters2@uq.edu.au (J.H.-P.); natalie.prow@qimrberghofer.edu.au (N.A.P.); kelly.baker1@uqconnect.edu.au (K.B.); thisun.piyasena@uqconnect.edu.au (T.B.H.P.); 2 Public Health Virology, Queensland Health Forensic and Scientific Services, Coopers Plain, Queensland 4108, Australia; E-Mail: carmel_taylor@health.qld.gov.au; 3 Protein Discovery Centre, QIMR Berghofer Medical Research Institute, Herston, Queensland 4029, Australia; E-Mails: ashok.rana@qimr.edu.au (A.R.); marcus.hastie@qimrberghofer.edu.au (M.L.H.); jeffrey.gorman@qimrberghofer.edu.au (J.J.G.)

**Keywords:** chikungunya virus, monoclonal antibodies, capsid protein, epitope mapping, linear B cell epitope, *C*-terminus, *N*-terminus

## Abstract

Chikungunya virus (CHIKV) is an arthropod-borne agent that causes severe arthritic disease in humans and is considered a serious health threat in areas where competent mosquito vectors are prevalent. CHIKV has recently been responsible for several millions of cases of disease, involving over 40 countries. The recent re-emergence of CHIKV and its potential threat to human health has stimulated interest in better understanding of the biology and pathogenesis of the virus, and requirement for improved treatment, prevention and control measures. In this study, we mapped the binding sites of a panel of eleven monoclonal antibodies (mAbs) previously generated towards the capsid protein (CP) of CHIKV. Using *N*- and *C*-terminally truncated recombinant forms of the CHIKV CP, two putative binding regions, between residues 1–35 and 140–210, were identified. Competitive binding also revealed that five of the CP-specific mAbs recognized a series of overlapping epitopes in the latter domain. We also identified a smaller, *N*-terminally truncated product of native CP that may represent an alternative translation product of the CHIKV 26S RNA and have potential functional significance during CHIKV replication. Our data also provides evidence that the *C*-terminus of CP is required for authentic antigenic structure of CP. This study shows that these anti-CP mAbs will be valuable research tools for further investigating the structure and function of the CHIKV CP.

## 1. Introduction

Alphaviruses are spherical, enveloped, positive-sense single-stranded RNA viruses responsible for several globally significant human and animal diseases. Alphavirus members include Sindbis virus (SINV), Semliki Forest virus (SFV), Ross River virus (RRV), the Western, Eastern and Venezuelan equine encephalitis viruses, as well as chikungunya virus (CHIKV), all of which are transmitted by mosquitoes. CHIKV infection is characterized by an onset of fever, headache, fatigue, nausea, myalgia and maculopapular rash, often followed by severe acute and/or chronic polyarthralgia [1,2,3,4]. Since its re-emergence in the early 2000s, CHIKV has been responsible for a succession of unprecedented outbreaks causing up to 6.5 million human infections in over 40 countries in East Africa, the Indian Ocean islands, several regions of South Asia, and most recently in Europe, Oceania and the Caribbean regions [1,5,6,7,8,9,10,11]. It has also been recently associated with severe disease manifestations, and mortality in some cases, largely amongst elderly patients with co-morbidities and the very young [12,13,14,15].

**Figure 1:**
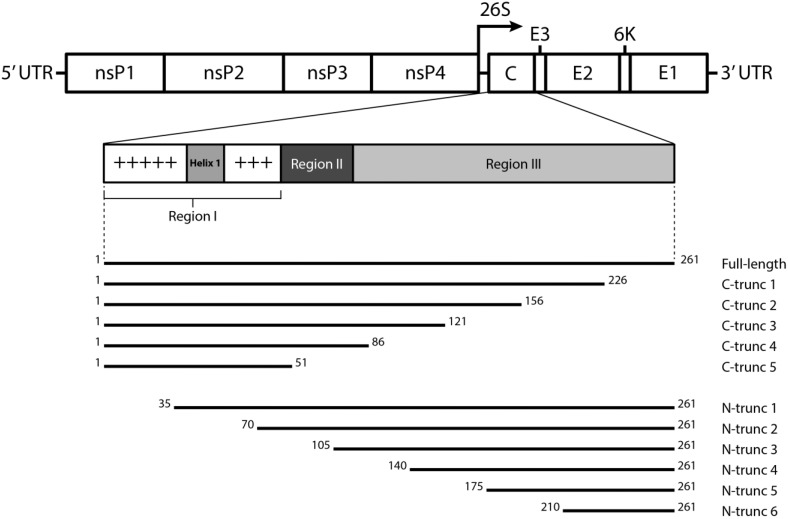
Schematic representation of the alphavirus capsid protein (CP) and the design of *N*- and *C*-terminal truncations in recombinant chikungunya virus (CHIKV) CP. The alphavirus CP is segmented into three separate regions, based on the prototype Sindbis virus (SINV) CP model—adapted from Hong *et al.* [20]. Region I has a high degree of positive charge associated with non-specific binding of the genomic viral RNA. Located within the same region, Helix 1, a sequence of uncharged amino acids, is suggested to be involved in the interactions during nucleocapsid core formation and its stabilization. Region II contains the minimum sequence required for specific RNA binding, while the *C*-terminal protease domain (region III) forms capsomeres in the nucleocapsid core and interacts with the E2 glycoprotein. The bottom part of the figure depicts the locations of the *C*- or *N*-terminal truncations within the full-length CP. Truncations were made in 35-amino acid increments.

Similar to other alphaviruses, CHIKV has a ~11.5 kb RNA genome that is capped at its 5′ end and polyadenylated at its 3′ end. The genome encodes four non-structural proteins, nsP1 to nsP4, and five structural proteins: capsid, E3, E2, 6K and E1 [16]. Studies have shown that the alphavirus capsid protein (CP) is multifunctional and plays a crucial role in the assembly and budding of alphaviruses. It is capable of self-cleavage prior to the recognition and binding of genomic RNA [17]. Apart from its primary role of forming the nucleocapsid, the CP has been shown to have inhibitive and/or regulative functions in regard to viral replication, as well as host and viral protein synthesis [18,19]. The CP of alphaviruses, as described in a SINV model, is organized into three separate regions—I, II and III—each with their respective functions [20]. As shown in Figure 1, the unconserved *N*-terminal domain of the alphavirus CP has a high degree of positive charge implicated in non-specific RNA binding, while the highly-conserved *C*-terminal region harbours a globular chemotrypsin-like serine protease and contains the binding site for the spike protein. A recent study by Thomas *et al.* [21] has also identified functional nuclear localization and export signals (NLS/NES) within the CHIKV CP. Furthermore, previous reports on the discovery of nucleolar targeting signals in SFV CP suggest that these signals are responsible for the karyophilic properties of the protein [22,23,24]. NLS sequences in forms of synthetic peptides have been used to demonstrate efficient transport of the CP into the nucleus of both higher and lower eukaryotic target cells [22]. Nonetheless, a functional role has yet to be attributed to this putative intracellular transport of the CP during infection.

The CP is a critical component of the assembly of alphaviruses. To date, alphaviral CP research is mainly based on models of SINV and SFV, and exact roles and/or functions of the CHIKV CP has largely been assumed to be similar to that of its closely-related viruses. However, the re-emergence of CHIKV and its potential threat to human health has demanded a more detailed understanding of the virus. Herein we report the use of a series of truncated recombinant proteins derived from the CHIKV CP, mass spec analysis of native CHIKV CP and competitive binding studies to map the binding sites of a panel of eleven monoclonal antibodies (mAbs) to antigenic domains at the *N*-terminal region and the *C*-terminal half of the protein.

## 2. Material and Methods

### 2.1. Cell and Virus Culture

C6/36 (*Aedes albopictus* mosquito) cells were propagated in RPMI 1640 supplemented with 2% fetal bovine serum. Cultures were passaged by dissociating the cell monolayer from the flask with trypsin/PBS and were incubated at 28 °C. Vero and COS-7L (African green monkey kidney) cell lines were cultured in DMEM and RPMI 1640, respectively, supplemented with 5% fetal bovine serum (FBS) during proliferation and 2% FBS for maintenance. Mammalian cells were passaged by dissociating the surface monolayer from the flask with trypsin/EDTA and were cultured at 37 °C with 5% CO_2_. Hybridoma cells were expanded in Hybridoma SFM (Gibco, Life Technologies, Carlsbad, CA, USA) with 20% FBS at 37 °C with 5% CO_2_, before being weaned off all FBS for the harvesting of mAbs in culture fluid. All cell cultures were supplemented with 50 U penicillin mL^−1^, 50 μg streptomycin mL^−1^ and 2 mM l-Glutamine (Gibco, Life Technologies).

Viruses used for the infection of C6/36 cells included CHIKV Mauritius strain (CHIKV_MAU_) (GenBank ID: EU404186) and RRV T48 strain (RRV_T48_) (GenBank ID: GQ433359). For harvesting of virus stocks, cells were infected at an M.O.I. of 0.1 for 1 h, washed thrice with PBS and incubated for a further 48–72 h before culture supernatants were clarified by centrifugation at 12,000× *g* for 10 min at 4 °C and stored at −80 °C. To obtain crude cell lysates, mock and/or virus-infected C6/36 cell monolayers were incubated in a similar manner before cells were rinsed in PBS and disrupted by sonication in the presence of BS9 lysis buffer (120 mM NaCl, 50 mM H_3_BO_3_, 1% Triton X-100 and 0.1% SDS, pH 9.0). Lysates were clarified as mentioned above and stored at −20 °C [25].

### 2.2. Cloning and Expression of CHIKV CP Truncations

CHIKV CP truncation constructs (Figure 1, bottom) were generated by amplifying the respective CP gene sequences from cDNA synthesized by reverse-transcription PCR of genomic RNA of CHIKV_MAU_, either by pairing the CHIKV Capsid Forward PCDNA primer with one of the CHIKV Capsid N1-6 Reverse PCDNA primers, or the CHIKV Capsid Reverse PCDNA primer with one of the CHIKV Capsid C1–5 Forward PCDNA primers (Table 1). Following that, inserts were ligated into a pcDNA3.1 (+) vector (Invitrogen) modified to express V5 and histidine tags at the *C*-terminus of the recombinant proteins. COS-7L cell transfection was performed using Lipofectamine 2000 (Invitrogen) according to manufacturer’s instructions. Cells were harvested 48 h post-transfection by addition of BS9 lysis buffer and clarified by centrifugation [26].

**Table 1:** Nucleotide sequences of CHIKV capsid truncation primers.

Primer code	Sequence (5′ to 3′)	Tm (°C)	Expected size (bp)
CHIKV Capsid F PCDNA	TATATAGCTAGCATGGAGTTCATCCCAACCCAA	76.7	-
CHIKV Capsid N1 R PCDNA	TATATAGGATCCGGCCACCACGCGTCCCT	72.3	678
CHIKV Capsid N2 R PCDNA	TATATAGGATCCGTGGTGCCAGTTGTAGTAC	55.3	573
CHIKV Capsid N3 R PCDNA	TATATAGGATCCCCGCTTAAAGGCCAGTTT	61.7	468
CHIKV Capsid N4 R PCDNA	TATATAGGATCCACCTTCGTGCTTGACTTC	57.8	363
CHIKV Capsid N5 R PCDNA	TATATAGGATCCCTGCTTCTTTTGATTTGTG	55.9	258
CHIKV Capsid N6 R PCDNA	TATATAGGATCCCAGTTTATTAACTGCTGAGATC	55.3	153
CHIKV Capsid R PCDNA	TATATAGGATCCACTCCACTCTTCGGCCCC	74.8	-
CHIKV Capsid C1 F PCDNA	TATATAGCTAGCATGAGGCAAGCTGGGCAAC	67.6	678
CHIKV Capsid C2 F PCDNA	TATATAGCTAGCATGAAGCAAAAACAACAGGC	74.8	573
CHIKV Capsid C3 F PCDNA	TATATAGCTAGCATGTGCATGAAAATCGAAAAT	60.7	468
CHIKV Capsid C4 F PCDNA	TATATAGCTAGCATGAAGGGGACCATCGATAA	67.7	363
CHIKV Capsid C5 F PCDNA	TATATAGCTAGCATGTCGAAGTTCACCCATGA	65.6	258

### 2.3. Immunofluorescence Assay (IFA)

Transfected COS-7L cells were fixed onto glass coverslips with 100% ice-cold acetone and incubated with selected mAbs in hybridoma culture fluid at a 1/20 dilution for 1 h at 37 °C. Coverslips were then washed and stained with Alexa Fluor 488-conjugated goat anti-mouse IgG (Invitrogen, Carlsbad, CA, USA) diluted 1:500 in blocking buffer (5% BSA in PBS) for 1 h at 37 °C, followed by Hoechst 33,342 stain (1:1000 in PBS, Invitrogen) for 5 min. Coverslips were mounted with ProLong Gold Anti-Fade reagent (Invitrogen) and imaged using a Zeiss LSM 510 META confocal microscope (Carl Zeiss AG, Oberkochen, Germany).

### 2.4. Western/Dot Blot

CHIKV antigens were prepared as transfected COS-7L cell lysates as described by Setoh *et al.* [27]. Reduction and carboxymethylation of antigens were carried out as previously described [28]. Briefly, lysates were diluted in Tris-HCl and reduced with 10 mM dithiothreitol (DTT). Samples were then degassed with streaming nitrogen and heated to 95 °C. The reduced lysates were cooled and iodoacetic acid was added before being subjected to a second round of degassing, followed by incubation at 37 °C in the dark. Antigens that were to be resolved on 4%–12% Bis-Tris precast SDS-PAGE gels (Invitrogen), were prepared in 4 × NuPAGE LDS sample buffer (Invitrogen) and heated to 95 °C for 5 min prior to electrophoresis. The separated proteins were then transferred onto Hybond C nitrocellulose membranes (Amersham) and immune-stained as previously described [25]. For dot-blotting, treated or untreated antigen samples were carefully spotted directly onto Hybond C nitrocellulose membranes and allowed to dry for 5–10 min. Membranes were then blocked with TENTC blocking buffer (0.05 M Tris-HCl pH 8.0, 1 mM EDTA, 0.15 M NaCl, 0.05% (*v/v*) Tween 20, 0.2% (*w/v*) casein) for 1 h at room temperature prior to the addition of primary antibodies diluted 1/20, unless otherwise stated, in blocking buffer. After incubation for another hour, membranes were washed thrice with 0.1% Tween-20 in PBS (PBS/T wash buffer) and bound antibodies were detected with a HRP-conjugated goat anti-mouse IgG diluted 1:4000 in blocking buffer. The blots were incubated for a further 1 h before being washed three times with PBS/T wash buffer. Finally, blots were developed in DAB substrate solution (1.5 mM 3,3′-diaminobenzidine, 0.06% (*v/v*) H_2_O_2_ in PBS, pH 7.2) for 15 min before reactions were terminated by rinsing with PBS or ddH_2_O.

### 2.5. Competitive Binding Enzyme-Linked Immunosorbent Assay (ELISA)

Competitive binding between anti-CHIKV CP mAbs were assessed as described previously [29]. Briefly, purified mAbs were biotinylated using the BiotinTag kit (Sigma-Aldrich, St. Louis, MO, USA), according to manufacturer’s instructions. Competitive binding ELISAs were performed in 96 well plates coated with lysates of CHIKV_MAU_-infected C6/36 cells diluted 1/500 in coating buffer (0.05 M sodium carbonate/bicarbonate, pH 9.6). After washing, a pre-defined optimal saturating concentration of each of the unlabelled mAbs was added for 1 h at 28 °C. Without washing, a pre-defined optimal non-saturating dilution of each biotin-labelled ‘competitor’ mAb (40 ng/mL) was added for 1 h at 28 °C. After washing six times with PBS/T, horseradish peroxidase (HRP)-conjugated streptavidin (Invitrogen) was added and incubated for 30 min. The wells were washed prior to incubation with ABTS substrate solution. Uninfected C6/36 cell lysates were used as coating antigen to determine background cut-off values, while 4G2, a pan-reactive mAb targeting the flavivirus envelope protein, was used as the negative antibody control.

### 2.6. Assessing Reactivity of mAbs to Synthetic Peptides

A series of 20-mer peptides, with 10-residue overlaps, encompassing the entire CHIKV CP were designed (Table 2) and commercially synthesized (Mocell Biotech, Hong Kong). Each peptide was coupled to carboxylated polystyrene beads using the Bio-Plex amine coupling kit (Bio-Rad, Hercules, CA, USA) according to the manufacturer’s instructions. The coupled beads were then multiplexed by diluting in microsphere immunoassay (MIA) buffer (1% BSA, 0.05% ProClin 300 (Supelco, Bellefonte PA, USA) in PBS) and distributed to give approximately 2500 of each beadset (~100 µL) per well of a MultiScreen filter plate (Merck Millipore, Billerica, MA, USA). Each anti-CHIKV CP mAb was then tested against the multiplexed beadsets by incubation for 45 min with shaking at room temperature. The wells were then washed three times with PBS/T wash buffer, prior to the addition of R-Phycoerythrin donkey anti-mouse IgG (Jackson ImmunoResearch, West Grove, PA, USA). Following that, the wells were incubated and washed as previously, after which the beads were resuspended in MIA buffer. The Bio-Plex 200 (Luminex, Bio-Rad, Austin, TX, USA) was then utilized to read the plate, measuring the mean fluorescence intensity for each beadset in their respective wells. mAb recognition of peptides was also assessed by ELISA and dot blot as previously described [29].

**Table 2:** Reactivity of mAbs to synthetic peptides.

No.	Sequence	Length	Protein *	Position	MIA	ELISA	Dot Blot
1.	MEFIPTQTFYNRRYQPRPWT	20	CHIKV CP	1–20	−	−	−
2.	NRRYQPRPWTPRPTIQVIRP	20	CHIKV CP	11–30	−	−	−
3.	PRPTIQVIRPRPRPQRQAGQ	20	CHIKV CP	21–40	−	−	−
4.	RPRPQRQAGQLAQLISAVNK	20	CHIKV CP	31–50	−	−	−
5.	LAQLISAVNKLTMRAVPQQK	20	CHIKV CP	41–60	−	−	−
6.	LTMRAVPQQKPRRNRKNKKQ	20	CHIKV CP	51–70	−	−	−
7.	PRRNRKNKKQKQKQQAPQNN	20	CHIKV CP	61–80	−	−	−
8.	KQKQQAPQNNTNQKKQPPKK	20	CHIKV CP	71–90	−	−	−
9.	TNQKKQPPKKKPAQKKKKPG	20	CHIKV CP	81–100	−	−	−
10.	KPAQKKKKPGRRERMCMKIE	20	CHIKV CP	91–110	−	−	−
11.	RRERMCMKIENDCIFEVKHE	20	CHIKV CP	101–120	−	−	−
12.	NDCIFEVKHEGKVTGYACLV	20	CHIKV CP	111–130	−	−	−
13.	GKVTGYACLVGDKVMKPAHV	20	CHIKV CP	121–140	−	−	−
14.	GDKVMKPAHVKGTIDNADLA	20	CHIKV CP	131–150	−	−	−
15.	KGTIDNADLAKLAFKRSSKY	20	CHIKV CP	141–160	−	−	−
16.	KLAFKRSSKYDLECAQIPVH	20	CHIKV CP	151–170	−	−	−
17.	DLECAQIPVHMKSDASKFTH	20	CHIKV CP	161–180	−	−	−
18.	MKSDASKFTHEKPEGYYNWH	20	CHIKV CP	171–190	−	−	−
19.	EKPEGYYNWHHGAVQYSGGR	20	CHIKV CP	181–200	−	−	−
20.	HGAVQYSGGRFTIPTGAGKP	20	CHIKV CP	191–210	−	−	−
21.	FTIPTGAGKPGDSGRPIFDN	20	CHIKV CP	201–220	−	−	−
22.	GDSGRPIFDNKGRVVAIVLG	20	CHIKV CP	211–230	−	−	−
23.	KGRVVAIVLGGANEGARTAL	20	CHIKV CP	221–240	−	−	−
24.	GANEGARTALSVVTWNKDIV	20	CHIKV CP	231–250	−	−	−
25.	SVVTWNKDIVTKITPEGAEEW	21	CHIKV CP	241–261	−	−	−
26.	SAAKHARKERNITGGHPVSR ^ǂ^	20	WNV_KUN_ NS5	38–57	+	+	+
27.	CTTVESHGNYSTQVGATQAG ^ǂ^	20	WNV_NY99_ E	146–165	+	+	+
28.	Full-length CHIKV CP	261	CHIKV CP	N/A	^#^ ND	+	+

* Each synthetic peptides was screened against all eleven anti-CHIKV CP mAbs, polyclonal anti-CHIKV antibodies, as well as 5H1 (anti-WNV_KUN_α3) and 17D7 (anti-WNV_NY99_ E) [29,30]; ^ǂ^ A “+” result represents reactivity with the peptide’s respective control mAb; ^#^ ND = not determined; MIA = microsphere immunoassay; Full-length CHIKV CP was reactive towards all eleven anti-CHIKV CP mAbs and polyclonal anti-CHIKV antibodies in ELISA and dot blot.

### 2.7. Immunoprecipitation of CHIKV CP

Protein pull-downs were performed on clarified lysates of CHIKV_MAU_-infected C6/36 cells using Dynabeads Protein G (Novex, Life Technologies) according to manufacturer’s instructions. Boiled and/or reduced samples were then resolved via SDS-PAGE as described above (see Section 2.4) prior to incubation of the gel in Coomassie blue stain (1% (*w/v*) Coomassie R250, 10% glacial acetic acid, 40% methanol in ddH_2_O) at room temperature for 30 min before being rinsed twice with destaining solution (10% glacial acetic acid, 40% methanol in ddH_2_O). The gel was then incubated in destaining solution overnight with rocking.

### 2.8. Mass Spectrometry Analysis

Individually stained bands were excised, reduced, alkylated and subjected to in-gel tryptic digestion as described in detail previously [31]. Acidified digests were subjected to NanoHPLC-MS/MS analysis using a nanoAcquity nanoHPLC system (Waters, Milford, MA, USA) interfaced with a linear ion-trap (LTQ)-Orbitrap Elite hybrid mass spectrometer (Thermo Fischer Scientific, Bremen, Germany). Digests were loaded onto a 5 μm Symmetry 180 μm × 20 mm C18 trap column (Waters) at 15 μL/min in 98% solvent A (0.1% (*v/v*) aqueous formic acid) and 2% solvent B (0.1% (*v/v*) formic acid in 100% acetonitrile) for 3 min at 22 °C then switched in-line with a pre-equilibrated analytical column (BEH130 C18 1.7 μm, 75 μm × 200 mm, Waters) at a flow rate of 0.3 μL/min and 98% solvent A, 2% solvent B. Peptides were separated at 35 °C using a sequence of linear gradients: starting from 5% B over 1 min, to 40% B over 29 min, and finally, to 95% B over 4 min, before holding the column at 95% B for a further 4 min. Eluates from the analytical column were then introduced into the LTQ-Orbitrap Elite throughout the entire run via a Nanospray Flex Ion Source (Thermo Fisher Scientific) containing a 10 μm P200P coated silica emitter (New Objective). Typical spray voltage was 1.8 kV with no sheath, sweep or auxiliary gases; heated capillary temperature was set to 275 °C. The LTQ-Orbitrap Elite was controlled using Xcalibur 2.2 SP1.48 software (Thermo Fisher Scientific) and operated in a data-dependent acquisition mode to automatically switch between Orbitrap-MS and ion trap-MS/MS. The survey full scan mass spectra (from m/z 380–1700) were acquired in the Orbitrap with a resolving power of 120,000 after accumulating ions to an automatic gain control (AGC) target value of 1.0 × 10^6^ charges in the LTQ. MS/MS spectra were concurrently acquired in the LTQ on the 20 most intense ions from the survey scan, using an AGC target value of 1.0 × 10^4^. Charge state filtering, where unassigned precursors and singly charged ions were not selected for fragmentation, and dynamic exclusion (repeat count 1, repeat duration 30 s, exclusion list size 500, exclusion duration 30 s) were used. Fragmentation conditions in the LTQ were: 35% normalized collision energy, activation q of 0.25, 10 ms activation time, and minimum ion selection intensity of 500 counts. Maximum ion injection times were 250 ms and 100 ms for survey full scans and MS/MS scans, respectively.

### 2.9. Data Analysis

Tandem mass spectra were processed using Proteome Discoverer (version 1.4, Thermo Fisher Scientific, San José, CA, USA) and submitted to Mascot (version 2.5.1, Matrix Science, London, UK). Fixed modification: carbamidomethyl-cysteine; variable modifications: deamidation (asparagine, glutamine) and oxidation (methionine). Enzyme: trypsin, 2 missed cleavages; MS tolerance 20 ppm; MSMS tolerance 0.6 Da using a database downloaded from UniprotKB on the 21 July 2014 consisting of the reference proteome for Aedes aegypti (UP000008820), chikungunya virus (strain 37997) (UP000008450), reference proteome chikungunya virus (strain S27-African prototype) with the sequence of the capsid protein added as a separate entry. Scaffold (4.4.1.1, Proteome Software, Portland, OR, USA, 2014) was used to validate Mascot protein identifications [32]. Scaffold probabilistically validates these peptide identifications using PeptideProphet [33]. A cut-off of 99% +2, +3 and 5 ppm error was used to validate the peptides.

## 3. Results

### 3.1. Anti-CHIKV CP mAbs Recognize Linear Epitopes

**Figure 2:**
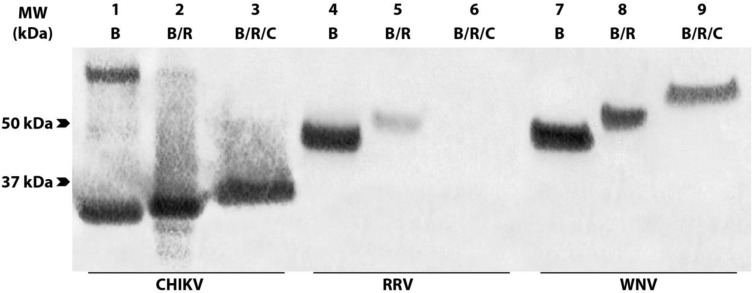
Reactivity of anti-CHIKV CP mAbs against boiled, reduced and/or carboxymethylated CHIKV cell lysates. Boiled (B), boiled and reduced (B/R) or boiled, reduced and carboxymethylated (B/R/C) lysates of CHIKV (lanes 1–3), RRV (lanes 4–6) and West Nile virus (lanes 7–9) were probed with antibodies 1.7B2 (anti-CP), RRG8 (anti-E1) and 17D7 (anti-E), respectively, in Western blot. RRV and West Nile virus lysates were included as controls to illustrate binding of reference antibodies known to recognize conformational (RRG8) and linear epitopes (17D7) respectively.

To determine whether a panel of eleven anti-CHIKV CP mAbs bound conformational or linear epitopes, antigens in the form of crude lysates derived from CHIKV-infected C6/36 cells were subjected to reduction with DTT and carboxymethylation of their free sulfhydryl groups so as to prevent the reformation of disulfide bonds. These antigens were separated by SDS-PAGE alongside boiled, unreduced, or boiled, reduced, uncarboxymethylated lysates prior to immunoblotting. Probing with each of the CP-specific mAbs, including representative antibody 1.7B2 (Figure 2), revealed that all eleven antibodies recognized CP under both unreduced (~34 kDa) and reduced (~35 kDa) conditions, even after carboxymethylation (~37 kDa), indicating that the anti-CHIKV CP mAbs recognized linear epitopes that are not dependent on disulfide bonds to provide secondary structure. The increase in molecular weight with each treatment is an indication of successful chemical modification due to the thiol-disulfide exchange during reduction and the addition of iodoacetic acid during S-carboxymethylation of cysteine residues within the CP.

Since the Western blot results indicated that all anti-CP mAbs recognized linear epitopes, we designed a series of 20-mer synthetic peptides, with 10-residue overlaps, spanning the entire CHIKV CP to fine-map the epitopes of the anti-CHIKV mAbs. However, none of the mAbs showed any reactivity towards the synthetic peptides in ELISA, dot blot or in MIA, despite the successful detection of all control peptides by their corresponding reference mAbs using these assays (see Table 2). This surprising result suggested that the binding of the CP-specific mAbs may require additional structure provided by post-translational modifications not found in synthetic peptides.

**Figure 3:**
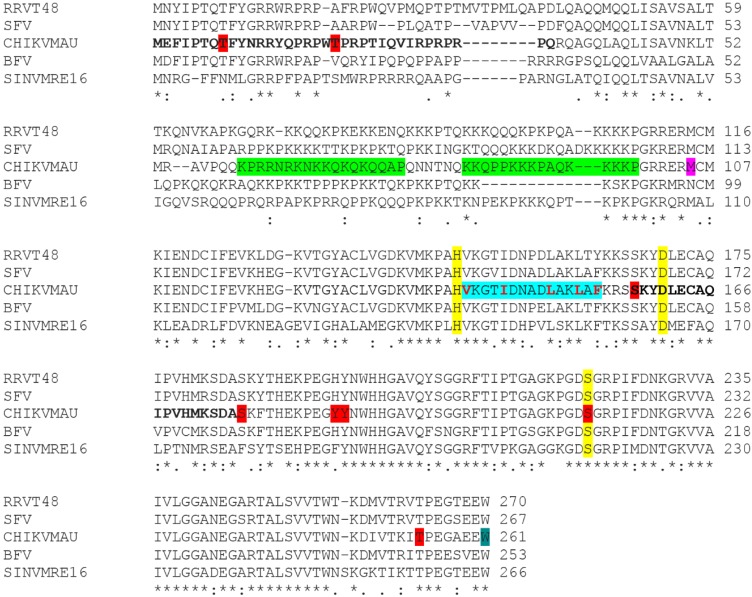
Capsid protein amino acid sequence alignment of closely-related alphaviruses. Asterisks (*), colons (:) and periods (.) indicate identical, conserved or semi-conserved substitutions, respectively. Residues highlighted in red represent potential phosphorylation sites predicted by NetPhos 2.0 (http://www.cbs.dtu.dk/services/NetPhos/), while yellow residues mark the conserved catalytic triad residues involved in the auto-proteolytic function of the CP. The teal-coloured tryptophan (position 261) is where the auto-cleavage occurs. Contributing amino acids of the predicted functional nuclear localization and export signals (NLS/NES) sequences are highlighted in green and blue, respectively. Red, bolded residues of the NES denote hydrophobic positions, indicating its similarity towards a non-classical NES sequence (Thomas *et al.*, 2013). The methionine (position 105) highlighted in magenta indicates the potential alternative translation initiation site of the *N*-terminally truncated form of CP. Putative mAb binding domains are in bold (group 1 mAbs: 157–175; group 2 mAbs: 1–35).

We have previously shown that these mAbs react with CP treated with PNGase F, indicating that *N*-linked glycosylation is not required, consistent with the absence of an *N*-linked glycosylation site and the cytoplasmic location of the protein [34]. Further analysis of the CHIKV CP sequence using a prediction software, NetPhos 2.0 [35], revealed the presence of several potential phosphorylation sites that may participate in the structure of epitope(s) recognized by these mAbs (Figure 3). However, attempts to demonstrate this by dephosphorylation of CHIKV CP prior to analysis by Western blot were inconclusive.

### 3.2. Expression of N- and C-terminally Truncated CHIKV CP

**Figure 4:**
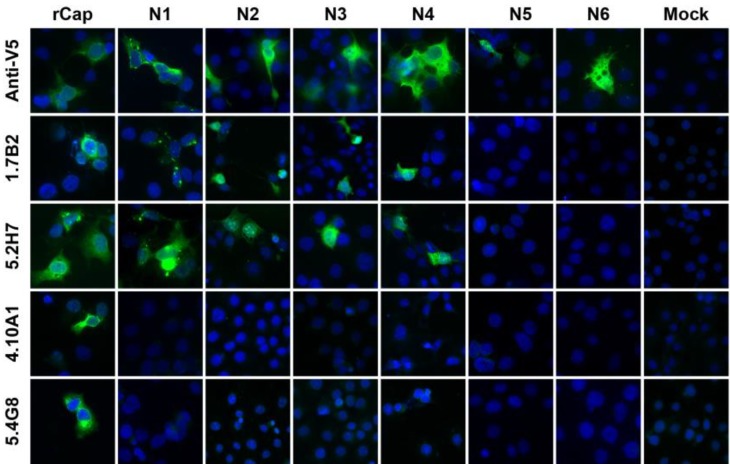
Monoclonal antibody reactivity by Immunofluorescence Assay (IFA) with acetone-fixed monolayers of COS-7L cells transfected with full-length rCap or N-truncated constructs. Cells were probed with respective mAbs before incubation with an anti-mouse Alexa Fluro 488 conjugate (green) and Hoechst 33,342 (blue) for nuclear staining. Two mAbs representing each group were chosen for this experiment: Group 1—1.7B2, 5.2H7; and Group 2—4.10A11, 5.4G8. Images were captured at 400× or 1000× magnification.

**Figure 5:**
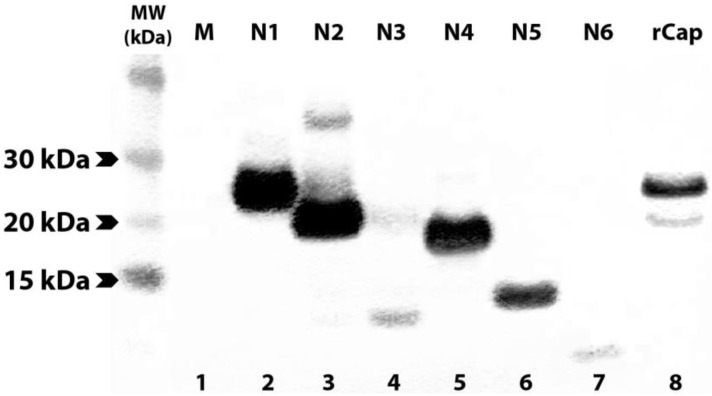
Western blot of recombinant full-length and N-truncated CP in lysates of transfected cells. Clarified lysates of transfected COS-7L cells were boiled and reduced with DTT prior to immunoblotting with anti-V5 mAb. Lane 1: mock-transfected COS-7L lysate; lanes 2–7: *N*1-6; and lane 8: full-length rCap.

In another approach to map the epitope(s) recognized by the anti-CP mAbs, recombinant full-length and a series of *N*- and *C*-terminally truncated CP sequences (Figure 1) were expressed in COS-7L cells. Successful expression of full length and truncated proteins *C*-terminally fused to a V5-His tag, was confirmed by detection with anti-V5 mAb in IFA (Figure 4, Figure S1). Most of the truncations were also recognized by anti-V5 mAb in Western blot (Figure 5, Figure S2), although the smaller protein bands were not successfully transferred to the nitrocellulose membrane due to limitation of the membrane pore size.

To identify the binding regions of these mAbs in CP, each antibody was subjected to Western blot analysis against each of the twelve truncated versions of CHIKV CP. Monoclonal antibodies 1.7B2, 4.1H11, 5.2H7, 5.5D11 and 5.5G9 (designated group 1), reacted with a series of the *N*-truncations (*N*1-4), as well as the full-length protein (rCp) (Table 3). This demonstrated that group 1 mAbs still recognized CP in the absence of the first 140 residues (see Figure 1) suggesting the epitope(s) they recognize resides in the *C*-terminal half of the protein. Failure of the group 1 mAbs to bind CP with larger truncations of between 175 (N5) and 210 residues (N6), conservatively placed their binding site between residues 140 and 210 in CP. Meanwhile, group 2 mAbs (4.8E2, 4.10A11, 5.1B2, 5.2F8, 5.4G8 and 5.5A11) were able to detect rCap but none of the *N*-terminally truncated proteins (Table 3). This suggested that all mAbs from group 2 bound the *N*-terminal region of the CP, since only the first 35 residues were missing from the smallest *N*-truncation (N1). Similar results were observed when selected group 1 and 2 mAbs were tested against the *N*-terminally truncated recombinant proteins in acetone-fixed transfected cells by IFA (Figure 4).

**Table 3:** Reactivity of anti-CHIKV mAbs against full-length, *C*- or *N*-terminally truncated versions of rCap in Western blot.

Monoclonal antibody	Group	Reactivity in Western blot											
		FL	N1	N2	N3	N4	N5	N6	C1	C2	C3	C4	C5
1.7B2	1	+	+	+	+	+	−	−	−	−	−	−	−
4.1H11	1	+	+	+	+	+	−	−	−	−	−	−	−
5.2H7	1	+	+	+	+	+	−	−	−	−	−	−	−
5.5D11	1	+	+	+	+	+	−	−	−	−	−	−	−
5.5G9	1	+	+	+	+	+	−	−	−	−	−	−	−
5.1B12	2	+	−	−	−	−	−	−	−	−	−	−	−
5.5A11	2	+	−	−	−	−	−	−	−	−	−	−	−
4.8E2	2	+	−	−	−	−	−	−	−	−	−	−	−
4.10A11	2	+	−	−	−	−	−	−	−	−	−	−	−
5.2F8	2	+	−	−	−	−	−	−	−	−	−	−	−
5.4G8	2	+	−	−	−	−	−	−	−	−	−	−	−

When all mAbs were tested against *C*-terminally truncated forms of CP, none of the antibodies from either group recognized any of the truncated proteins in IFA (Figure 4) or Western blot (Table 3) despite their recognition by the anti-V5 antibody, suggesting that the *C*-terminal region of CP might be required for authentic antigenic structure of CP. This was confirmed by the reactivity of mouse polyclonal anti-CHIKV antibody with rCap and *N*-terminally truncated proteins (*N*1–4) in Western blot but none of the *C*-terminally truncated proteins (Figure S3).

### 3.3. Group 1 mAbs Bind Native N-terminally Truncated CP in CHIKV Lysates

**Figure 6:**
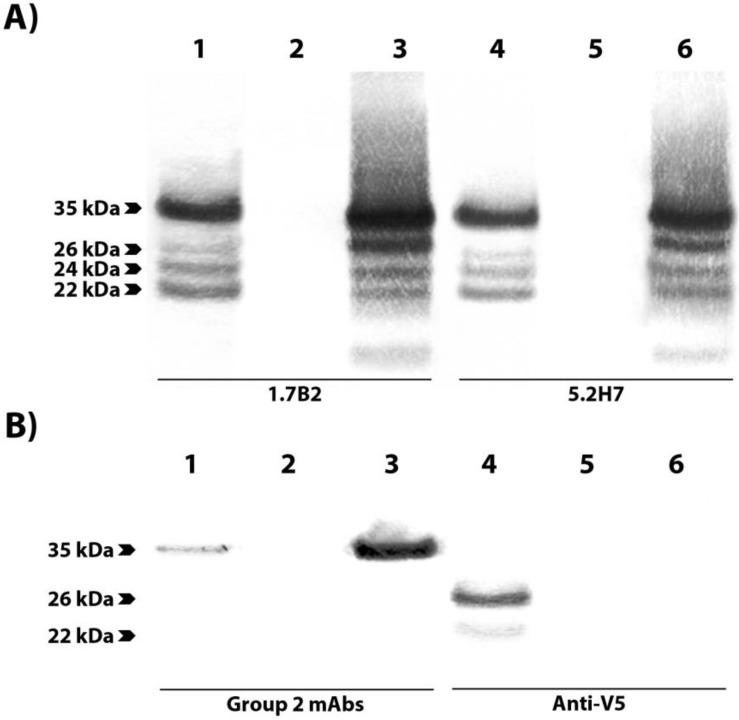
Reactivity of anti-CP mAbs with full length and truncated versions of native and recombinant CP. Reactivity of mAbs 1.7B2 (lanes A1–3), 5.2H7 (lanes A4–6), cocktail of group 2 mAbs (lanes B1–3) and anti-V5 mAb (lanes B4–6) in Western blot against boiled and reduced lysates of COS-7L cells expressing rCap (lanes 1 and 4), mock-transfected COS-7L cells (lanes 2 and 5), and CHIKV-infected C6/36 cells (lanes 3 and 6).

IFA and Western blot analysis of *N*-terminally truncated recombinant CP indicated that group 1 mAbs recognized a region in the *C*-terminal half of the CHIKV CP while group 2 mAbs bound the *N*-terminal region. We also observed that, in addition to detecting the rCap in lysates of CHIKV-infected cells (~35 kDa), group 1 mAbs also detected smaller, truncated versions of native CP (sCP) ranging from ~15–30 kDa (Figure 6A). In contrast, antibodies from group 2 only recognized full-length CP in these lysates (Figure 6B, Figure S4).

**Figure 7:**
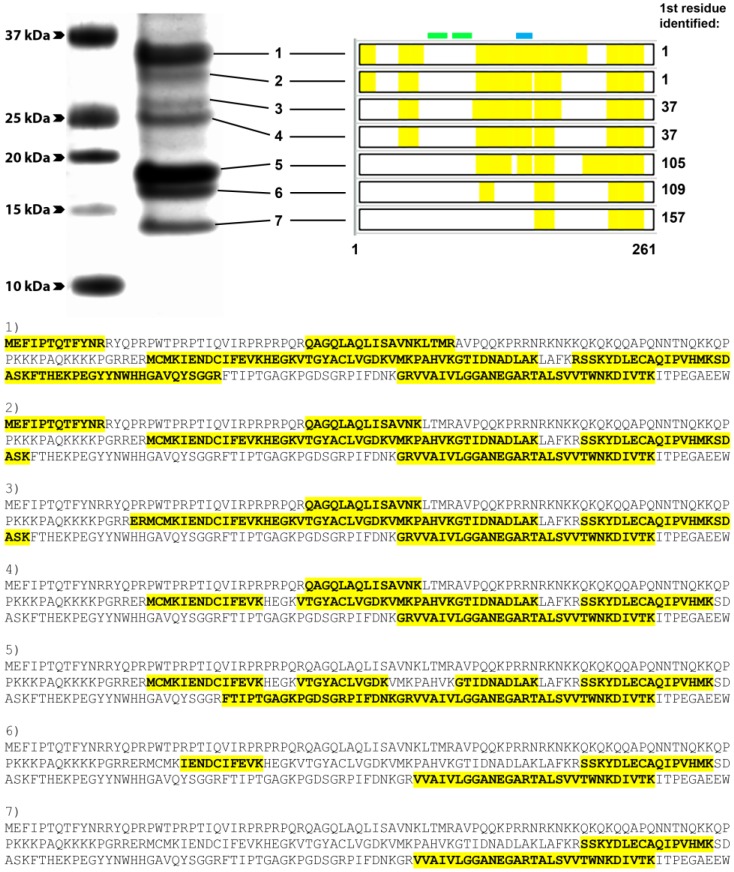
Coverage of the CHIKV capsid protein by peptides identified via mass spectrometry analysis of different bands excised from SDS-PAGE of boiled and reduced proteins immunoprecipitated by a group 1 mAb, 1.7B2. Indicated in green and blue are the predicted positions of the CHIKV CP NLS and NES, respectively. Sequences for peptides identified within each respective CHIKV CP band (1–7) are bolded and highlighted in yellow.

To determine the identity of the truncated native versions of CP, capsid proteins were immune-precipitated from CHIKV-infected lysates with protein G beads coupled to mAb 1.7B2 (group 1). Resulting pull-downs were resolved on SDS-PAGE and selected Coomassie-stained CPs were analysed by mass spectrometry. These analyses showed that all species of CP recognized by mAb 1.7B2, including the rCap, contained the same *C*-terminal peptide (Figure 7). However, as the CP molecules were truncated, detection of the *N*-terminus region was progressively lesser, with observations that peptides toward the *N*-terminal side of the protein, that were previously identified in larger-sized bands, were missing. No smaller molecules of CP were precipitated, which was consistent with the binding pattern of 1.7B2 and other group 1 mAbs to *N*-terminally-truncated recombinant CPs; binding to *N*1 to N4 (35–140 residues removed) but not N5 and N6 (175 and 210 residues removed). Together, these data confirm that the epitopes recognized by 1.7B2, and other group 1 mAbs, were located between residues 140 and 210 in the *C*-terminal half of CP. Furthermore, the lack of recognition of native CP truncated by 36 or more residues from the *N*-terminus by group 2 mAbs, also supports their binding to an *N*-terminal domain—within the first 35 amino acids—of the protein.

### 3.4. Group 1 mAbs Target a Series of Overlapping Epitopes on CP

**Figure 8:**
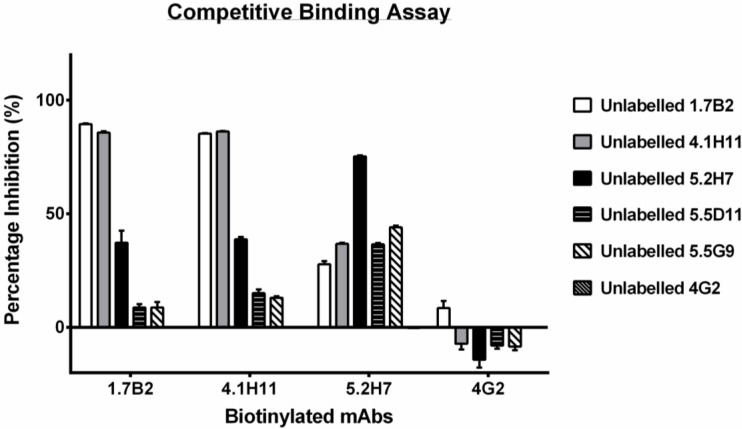
Competitive binding profiles of CHIKV CP-specific mAbs in ELISA. Antigens in lysates of CHIKV_MAU_-infected Vero cells were adsorbed to 96-well plates at a 1/500 dilution prior to incubation with a saturating dilution of purified, unlabelled anti-CP mAbs. Without washing, non-saturating dilutions of biotinylated mAbs were then added as ‘competitor’ antibodies to respective wells. The mean absorbance reading (OD_405nm_) of four replicates were plotted with bars showing standard error of mean (SEM). 4G2 is a control mAb specific to the E protein of flaviviruses. Assay was optimized to obtain complete inhibition of each biotinylated mAb by its homologous unlabelled competitor.

**Figure 9:**
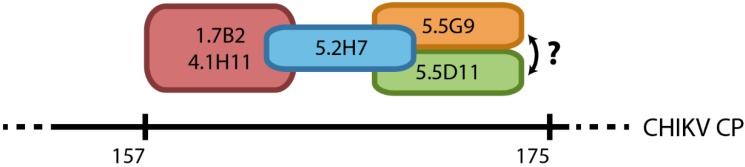
Schematic representation of proposed spatial relationship of epitopes recognized by group 1 anti-CP mAbs. Antibodies in the same coloured circle illustrates two-way inhibition, with overlaps representing one-way inhibition amongst mAbs tested. While it is safe to assume that the sequence of overlapping should be fairly accurate since the group 1 mAbs do recognize linear epitopes, the order of epitopes might be inversed. Furthermore, it is highly likely that mAbs bind from residues 157–175, however, a more conservative estimate would be from position 140–210.

To further define the binding sites of the mAbs from group 1, and determine the topology of their epitopes in the *C*-terminal half of CP, representative group 1 antibodies were tested in a competitive binding assay against one another using ELISA. Three of the five mAbs in this group (1.7B2, 4.1H11 and 5.2H7) were successfully biotinylated and subsequently assessed for competition against saturating concentrations of each of the five unlabelled mAbs to establish the degree of inhibition exhibited for each pairing. MAbs 1.7B2 and 4.1H11 showed complete two-way inhibition with each other indicating they bind to the same or highly adjacent epitopes (Figure 8). Partial two-way inhibition of both these mAbs with mAb 5.2H7 was also observed, suggesting that the epitope recognized by 5.2H7 is slightly different to the former pair but in the same spatial domain. The partial one-way inhibition of 5.2H7 by the unlabelled mAbs 5.5D11 and 5.5G9, but not by 1.7B2 and 4.1H11, suggests a continuum of overlapping epitopes (Figure 9). The competitive binding result also confirms that these antibodies bind to the same region of the CP.

## 4. Discussion

In this study, we used a series of truncated recombinant proteins to map the binding sites of a panel of CHIKV CP-specific mAbs produced in a previous study [34]. The binding patterns of the mAbs to recombinant truncated CPs in Western blot and IFA allowed us to deduce that these antibodies bound two regions of CP; one domain in the *C*-terminal half of the protein between residues 140 and 210 (group 1 mAbs), and a second putative domain likely located within the first 35 amino acids of the *N*-terminus of CP (group 2 mAbs). Mass spectrometry analysis of native, *N*-terminally truncated CP species in CHIKV-infected cell lysates that were recognized by group 1 but not group 2 mAbs, further supported the proposed location of the two binding domains of these mAbs. The binding of all group 1 mAbs to a defined region between residues 140 and 210 in the *C*-terminal half of CP was further indicated by the competitive binding experiments, demonstrating that these mAbs recognized a series of overlapping epitopes representing a continuum of binding sites. The proposed spatial relationship of epitopes recognized by the group 1 mAbs is schematically presented in Figure 9. The putative binding domain for the group 2 mAbs was indicated to be in the first 35 residues due to their inability to bind the smallest N-truncation in IFA and Western blot, and a lack of reactivity of these mAbs to native species of *N*-truncated CHIKV CP, as revealed by the mass spectrometry data. However, without direct evidence of group 2 mAbs binding to this region, the location of this domain requires further confirmation.

**Figure 10:**
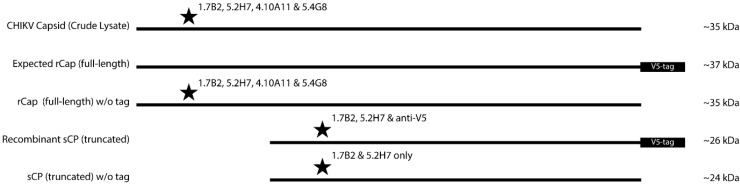
Schematic representation of different sizes of CP observed in Western blot. Stars represents the mAbs that are reactive toward that particular protein band. To obtain sizes of protein bands, a calibration curve was constructed by semi-logarithmically plotting the relative mobility values of the molecular weight standards against their known molecular weights. The relative mobility of each target protein was then utilized to estimate their molecular weight. sCP is truncated at the *N*-terminus.

Another interesting finding to emerge from this study was the presence of truncated CHIKV CPs in preparations of both native and recombinantly-expressed protein. Truncated fragments of CHIKV CP, displaying sizes similar to those observed in this study, have previously been identified in cells expressing recombinant CHIKV CP from a baculovirus vector, as well as cells infected with a recombinant measles vaccine expressing CHIKV virus-like particles [36,37]. This uncharacterized minor product of CP has also been documented in other alphaviruses such as WEEV and SINV [38,39,40]. Based on the observations made and data obtained in this study, a schematic of the different sized CP bands derived from molecular weight analysis of CP from both crude lysates of CHIKV-infected C6/36 cells and recombinantly expressed versions, was constructed (Figure 10). This suggests that the *N*-terminally-truncated sCP is approximately 10–11 kDa smaller than its full-sized counterpart and is truncated by ~100 residues from the *N*-terminus. This was confirmed by successful retention of the *C*-terminally inserted V5-His tag and the mass spectrometry analysis that the *N*-terminal peptide identified in the major native species of sCP in CHIKV-infected cell lysates began with the methionine at position 105 in CHIKV CP. This also corresponds with a predicted alternate translation initiation site at the third AUG of the 26S RNA in the alphavirus structural polyprotein, previously observed in a SINV mutant [41].

Studies with New World alphaviruses (e.g., Western/Eastern/Venezuelan equine encephalitis viruses) have shown that CP mediates shutdown of host cell transcription [19]. In contrast, the viral nsP2, not CP, appears to mediate this role in the Old World alphaviruses (e.g., Sindbis virus, Semliki Forest, Ross River virus) [42]. However, there is no published evidence that CHIKV, considered an Old World alphavirus, also uses the latter mechanism. Old World alphaviruses have evolved a downstream loop (DLP) in the 26S subgenomic RNA that stalls the ribosome at the first AUG, allowing acquisition of specific initiation factors and high level translation of viral protein in an environment where host gene translation in the infected vertebrate cell is suppressed due to eukaryotic initiation factor 2 alpha (eIF-2α) phosphorylation. The lack of a DLP-like structure in CHIKV is consistent with a recent report that CHIKV nsP4 prevents eIF-2α phosphorylation during infection and negates the need for a DLP enhancer and ribosome stalling at the first AUG. These findings also fit with our observations that an *N*-terminally truncated form of CP is produced in the CHIKV-infected cell, consistent with leaking ribosome scanning [43] and alternative translation initiation from the conserved 3rd AUG in the 26S subgenomic RNA (codon 105 in the capsid gene).

Our initial findings that all the anti-CP mAbs included in this study recognized reduced and carboxymethylated antigens in Western blot, indicated they bound to linear or continuous epitopes independent of tertiary structure. Thus, it was surprising that attempts to further define the binding sites of these antibodies using a series of 20-mer synthetic peptides with 10-mer overlaps covering the entire CHIKV CP were unsuccessful. None of the mAbs showed any reactivity towards the peptides when adsorbed to the solid phase in ELISA, bound onto nitrocellulose membranes in dot blot, or when presented on microspheres in a Luminex-based assay. The successful recognition of control peptides, synthesized in the same batch, by their corresponding mAbs in each assay indicated that incorrect synthesis was not likely to be the problem. This suggested that the epitopes on CHIKV CP required additional structures for mAb recognition, potentially post-translational modifications such as phosphorylation or glycosylation, as described in previous studies [44,45]. The CP of alphaviruses are known not to harbour any *N*-linked glycosylation, which was previously confirmed by endoglycosidase digestion [34]. However, eight potential sites of phosphorylation have been predicted in CHIKV CP based on its amino acid sequence, with two potentially phosphorylated residues at positions 8 and 20, both in the proposed binding region of group 2 mAbs, and one at residue 158 in the putative binding domain of group 1 mAbs (Figure 3).

The lack of reaction of all mAbs and polyclonal antisera to *C*-terminally truncated recombinant CP, despite their clear detection by anti-V5 in Western blot and IFA, was also unexpected considering the apparent linear nature of their epitopes. This suggests that the *C*-terminal residues may be crucial to the antigenic structure of CHIKV CP, either by facilitating phosphorylation of residues in the binding sites as referred to above, or by stabilizing the overall structure of CP and exposing these epitopes on the surface of the protein, allowing access for antibody binding. Both of these hypotheses fit well with the data obtained in this study, including the failure of the antibodies to recognize the synthetic peptides; the 20-mer peptides predicted to contain the putative binding sites (peptides no. 1–4 for group 2 mAbs; no. 15–18 for group 1 mAbs) would also lack the hypothesized *C*-terminal peptides of CP. Future studies to resolve this question and further define the binding sites of the anti-CP mAbs, could include the re-construction of rCap with variable internal deletions and the retention of the *C*-terminal residues.

## 5. Conclusions

In summary, we have mapped the binding sites of a panel of anti-CHIKV CP mAbs to two proposed domains. Group 2 mAbs recognize a putative domain between residues 1–35, while group 1 mAbs bind a region between residues 140–210; the latter targeting a series of overlapping epitopes. We also provided evidence that the *C*-terminus of CP is required for the authentic antigenic structure of the protein—and thus antibody binding—and have identified a smaller species of CP in CHIKV-infected cells that may represent an alternative translation product of the viral 26S RNA. Our epitope mapping studies have identified novel structural properties of the CHIKV CP and characterized useful reagents for their further investigation. A better understanding of the structure of CP will provide valuable insight into the multifunctional role of this protein in CHIKV replication, as well as that of numerous other alphaviruses.

## Supplementary Files

Supplementary File 1
