# Supplementary material for: The Chikungunya Virus Capsid Protein Contains Linear B Cell Epitopes in the N- and C-Terminal Regions that are Dependent on an Intact C-Terminus for Antibody Recognition
Source: Viruses. 2015 Jun 8;7(6):2943–64. doi: 10.3390/v7062754 (PMC4488721; doi:10.3390/v7062754)
Supplement: Supplementary File 1 [file viruses-07-02754-s001.pdf]

Supplementary Information

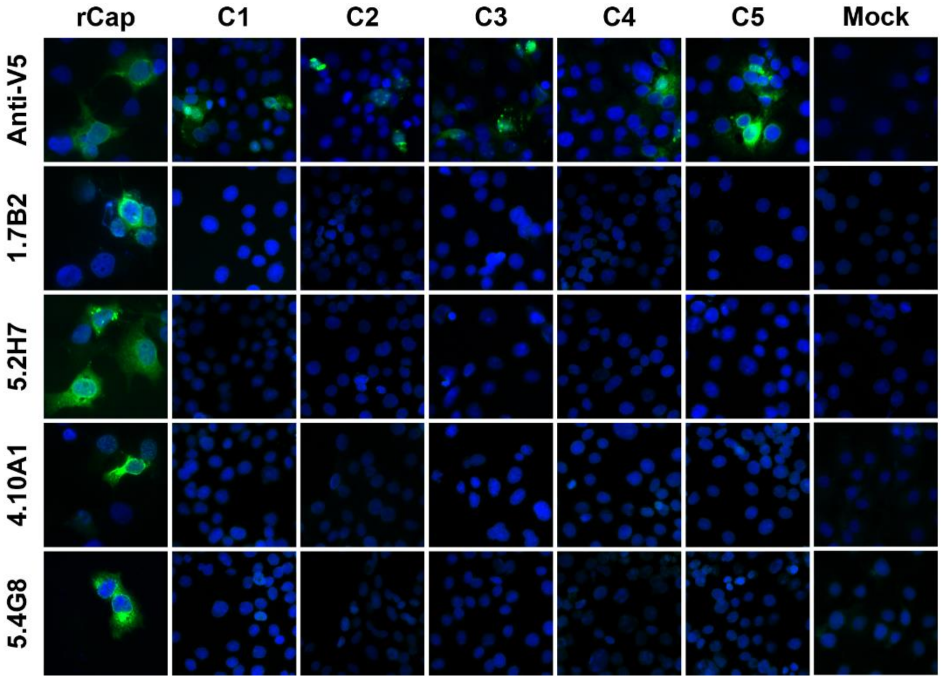

Figure S1. Monoclonal antibody reactivity by IFA with acetone-fixed monolayers of COS-7L cells transfected with full-length rCap or C-truncated constructs. Cells were probed with respective mAbs before incubation with an anti-mouse Alexa Fluro 488 conjugate (green) and Hoechst 33,342 (blue) for nuclear staining. Two mAbs representing each group were chosen for this experiment: Group 1—1.7B2, 5.2H7; and Group 2—4.10A11, 5.4G8. Images were captured at 400 × or 1000 × magnification.

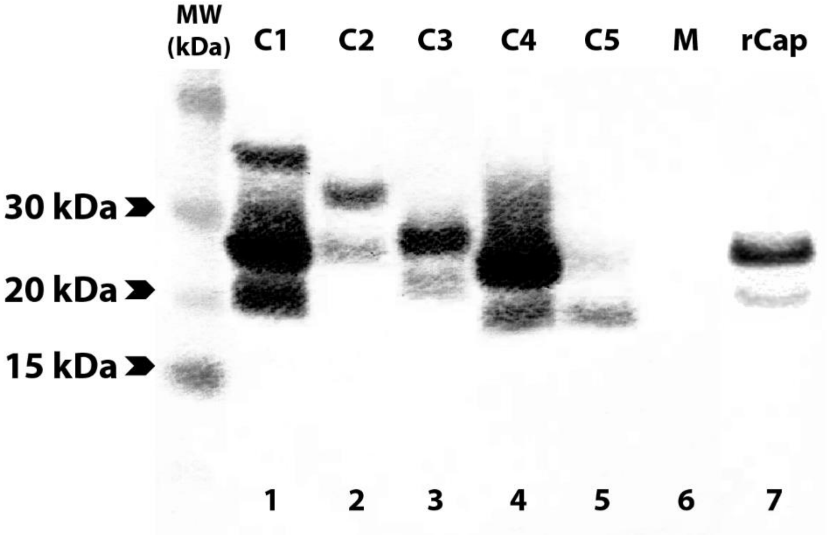

Figure S2. Western blot of recombinant full-length and C-truncated CP in lysates of transfected cells. Clarified lysates of transfected COS-7L cells were boiled and reduced with DTT prior to immunoblotting with anti-V5 mAb. Lane 1: mock-transfected COS-7L lysate; lanes 2–6: C1–5; and lane 7: full-length rCap.

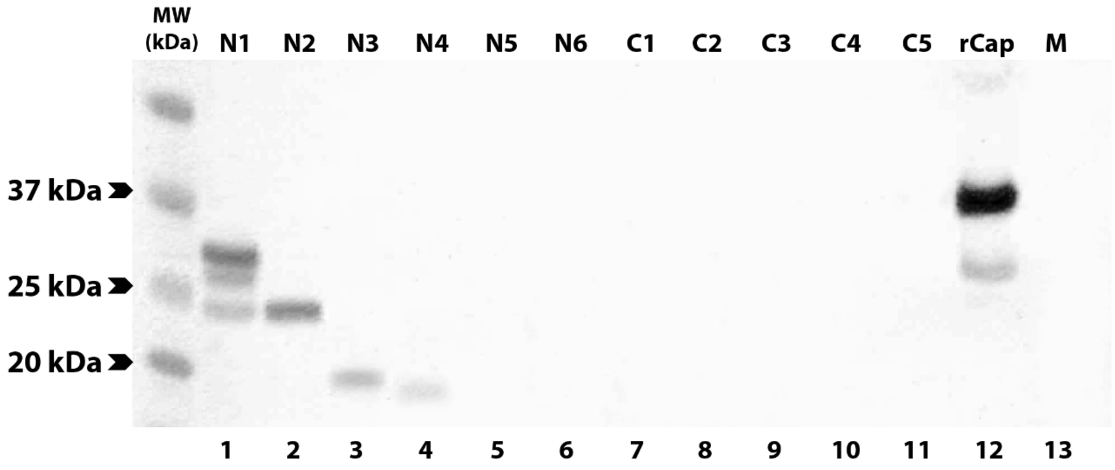

Figure S3. Reactivity of anti-CHIKV polyclonal mouse sera against recombinant CP truncations. Reactivity of polyclonal antibodies against N1-6 (lanes 1–6), C1–5 (lanes 7–11), rCap (lane 12), and mock-transfected COS-7L lysates (lane 13) in Western blot.

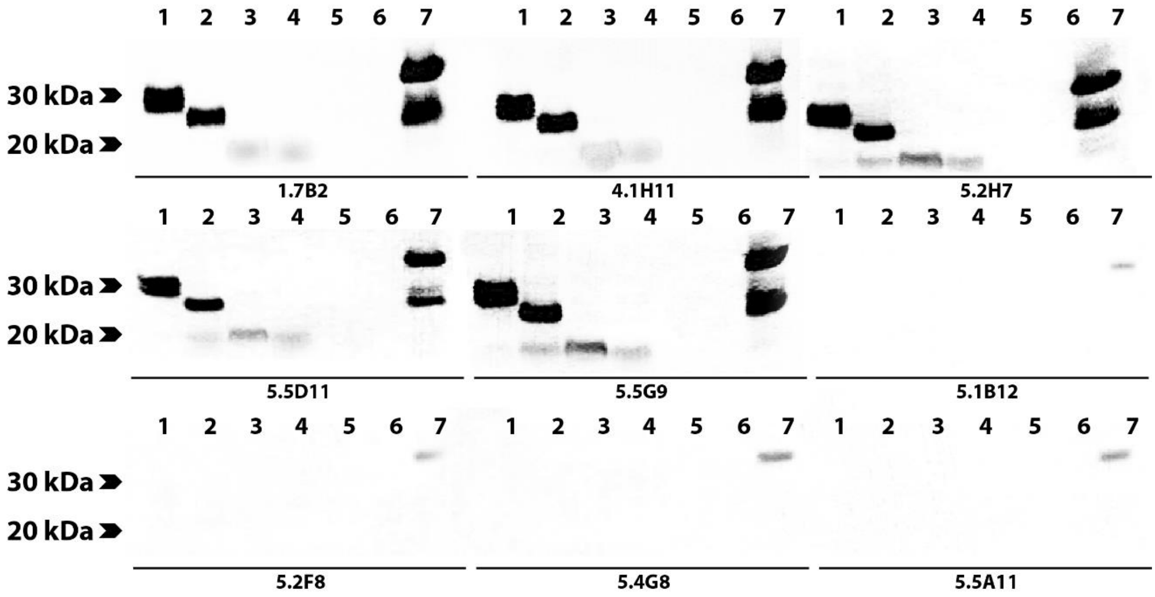

Figure S4. Reactivity of anti-CP mAbs with N-truncations and rCap in Western blot. CP-specific mAbs were tested for their reactivity against N1–6 (lanes 1–6) and rCap (lane 7).
